# The relative value of Pre-Implementation stages for successful implementation of evidence-informed programs

**DOI:** 10.1186/s13012-023-01285-0

**Published:** 2023-07-21

**Authors:** Zoe M. Alley, Jason E. Chapman, Holle Schaper, Lisa Saldana

**Affiliations:** Oregon Social Learning Center, 10 Shelton McMurphey Blvd, Eugene, OR 97401 USA

**Keywords:** Implementation fidelity, Stages of implementation completion, Pre-Implementation

## Abstract

**Background:**

Most implementations fail before the corresponding services are ever delivered. Measuring *implementation process fidelity* may reveal when and why these attempts fail. This knowledge is necessary to support the achievement of positive implementation milestones, such as delivering services to clients (program start-up) and competency in treatment delivery. The present study evaluates the extent to which implementation process fidelity at different implementation stages predicts achievement of those milestones.

**Methods:**

Implementation process fidelity data—as measured by the Stages of Implementation Completion (SIC)—from 1287 implementing sites across 27 evidence-informed programs were examined in mixed effects regression models with sites nested within programs. Implementation process fidelity, as measured by the proportion of implementation activities completed during the three stages of the SIC Pre-Implementation phase and overall Pre-Implementation (Phase 1) and Implementation (Phase 2) proportion scores, was assessed as a predictor of sites achieving program start-up (i.e., delivering services) and competency in program delivery.

**Results:**

The predicted probability of start-up across all sites was low at 35% (95% CI [33%, 38%]). When considering the evidence-informed program being implemented, that probability was nearly twice as high (64%; 95% CI [42%, 82%]), and 57% of the total variance in program start-up was attributable to the program. Implementation process fidelity was positively and significantly associated with achievement of program start-up and competency. The magnitude of this relationship varied significantly across programs for Pre-Implementation Stage 1 (i.e., Engagement) only. Compared to other stages, completing more Pre-Implementation Stage 3 (Readiness Planning) activities resulted in the most rapid gains in probability of achieving program start-up. The predicted probability of achieving competency was very low unless sites had high scores in both Pre-Implementation and Implementation phases.

**Conclusions:**

Strong implementation process fidelity—as measured by SIC Pre-Implementation and Implementation phase proportion scores—was associated with sites’ achievement of program start-up and competency in program delivery, with early implementation process fidelity being especially potent. These findings highlight the importance of a rigorous Pre-Implementation process.

Contributions to the Literature

- Despite widespread enthusiasm for evidence-informed programs (EIP), most EIP implementations fail. Measuring *implementation process fidelity* during the Pre-Implementation and Implementation Phases—via the Universal Stages of Implementation Completion (SIC)—could tell us why.
- Sites with high Pre-Implementation fidelity were more likely to deliver EIP services. Implementation fidelity (later in the implementation process) was positively and significantly associated with achieving competency in program delivery, but only if sites had high Pre-Implementation fidelity.
- To support implementation of EIPs, funders and allies of EIP adoption should accommodate the resources necessary for sites to conduct strong Pre-Implementation.

## Background

Although enthusiasm is high for integrating evidence-informed programs into standard practice [1, 2], over 60% of implementation efforts fail before delivering services to clients [3]. This identifies a clear need. To address it—and to benefit the individuals in need of evidence-informed services—strong implementation methods are essential [4].

### The role of implementation process fidelity

The role of *implementation process* fidelity—fidelity to the complete process of implementing an evidence-based program—mirrors the role of *intervention* fidelity. Traditionally, evidence-informed programs are developed through a series of tests, beginning with efficacy (controlled circumstances), then effectiveness (realistic circumstances), and then implementation (real-world circumstances). More recently, hybrid effectiveness-implementation trials have enabled simultaneous testing, with the aim of making evidence-informed programs available more quickly [5, 6]. Across this range of tests, a core component is *intervention* fidelity—that is, delivery of the intervention in the manner intended. More specifically, intervention fidelity is defined as adherence to key treatment components, where those components are delivered with competence [7, 8]. If an intervention is delivered with poor fidelity—that is, if the active treatment components are never delivered or delivered poorly—the intervention may fail to produce its intended outcomes. Therefore, to promote the successful delivery of an intervention, measurement of intervention fidelity is essential.

In a similar way to *intervention* fidelity, it is possible to consider *implementation process* fidelity. Whereas intervention fidelity focuses on the actual delivery of the intervention (e.g., from provider to client/patient), implementation process fidelity begins earlier, focusing on the complete process of implementing the evidence-informed intervention program in the intended service delivery setting. Just as there are specific intervention activities involved in administering an intervention (e.g., drafting an avoidance hierarchy with clients), there are specific implementation activities involved in implementing an evidence-informed program (e.g., facilitating community partner meetings with referring agencies). Completion of those activities as intended can be thought of as *implementation process fidelity*. A strong implementation process is critical for achieving key implementation milestones [4], for example, successfully initiating service delivery to clients. Measurement of implementation process fidelity has the potential to inform implementation strategies and explain implementation outcomes. Such measurement may be a critical consideration for reducing the well-known gap between knowledge and practice [9].

According to the ubiquitous EPIS framework, the implementation process is characterized by four phases: Exploration, Preparation, Implementation, and Sustainment (EPIS; [10]). Not all implementing sites complete activities in every phase. For example, a site may attempt to offer services without any Preparation, or after offering services, they may cease monitoring the program and complete no activities in Sustainment. Such departures from the implementation process—that is, low implementation process fidelity—may impact the achievement of implementation milestones. Measuring implementation process fidelity allows for this to be tested with implementation milestones spanning the implementation process, such as program start-up and competency in program delivery. An implementing site has achieved the early indicator of implementation success, *program start-up,* when they first deliver the corresponding program’s services to clients/patients. A site has achieved *competency in program delivery—*a long-term indicator of implementation success*—*when they can demonstrate that they consistently deliver those services as intended, with the necessary infrastructure and support for sustainment. Measuring implementation process fidelity across the EPIS framework can identify what activities in what phases support these implementation outcomes, thus increasing the potential for successful implementations and broadening the reach of evidence-informed programs.

### Pre-Implementation: a critical period

There is some evidence that implementation process fidelity during the initial phases of implementation—during Exploration and Preparation—may be particularly important for achieving later implementation milestones. In other words, eventual implementation success may depend, in part, on implementation process fidelity during the earliest parts of the implementation—i.e., Pre-Implementation [11]. This is supported by multiple studies, which have found that sites with higher implementation process fidelity early-on were more likely to make it to the point of delivering services to clients [12–15]. Additionally, in a descriptive study of 23 implementation attempts [16], sites that eventually became competent in delivering the intervention program were those that—early-on—had 100% implementation process fidelity. In contrast, of sites that did not reach competency in program delivery, the average level of implementation process fidelity during Pre-Implementation was only 47%. Other descriptive research has found that, among sites not succeeding with implementation, most discontinue during Pre-Implementation [3]. Finally, an implementation strategy targeting Pre-Implementation fidelity (i.e., social-cognitive pre-implementation enhancement strategies; SC-PIES) whereby teachers’ motivation and perception of the uptake of the EIP was targeted through training about the value of the intervention and committing to its use, was associated with higher teacher fidelity and improved behavioral outcomes for youth whose teachers received the intervention [17]. Together, these findings suggest that Pre-Implementation fidelity may lay the groundwork for achieving later success. However, these studies have limitations. Most have focused on implementation attempts for only one or two EIPs. This leaves questions about the consistency or variability of findings across a wider range of programs. Likewise, due to sample size, many studies including evaluations of later implementation outcomes, such as achieving competency in program delivery, have been restricted to descriptive evaluations. To address these limitations, a large sample of implementing sites across various EIPs is necessary.

### The present study

A site’s early implementation process fidelity may support achievement of both proximal and distal implementation milestones. The Stages of Implementation Completion (SIC)—which measures implementations with a widely used, web-based implementation process fidelity system [18]—provides an opportunity to investigate this association across EIPs. The SIC measures both implementation process fidelity and important implementation milestones, such as program start-up and achievement of competency in program delivery. The unit of measurement—rather than being a practitioner or service recipient—is an individual site (e.g., a clinic or facility) that is attempting to implement an EIP. Consistent with the EPIS framework, the SIC divides the implementation process into multiple phases: Pre-Implementation (EPIS Exploration and Preparation phases), Implementation, and Sustainment.

The SIC originally was developed to compare implementation process fidelity across two sites during a head-to-head trial of competing multicomponent implementation strategies [12] and has been adapted or customized to track implementation process fidelity for more than 2,000 sites [19] and 70 evidence-informed programs (e.g., [20–22]). For example, Aalsma and colleagues [23] are utilizing the SIC to monitor the implementation process of the implementation of a multi-system, multi-component intervention to treat substance use disorders in youth involved in the juvenile justice system. In a very different context, Dubowitz and colleagues [24] are using the SIC to monitor their evaluation of two training strategies for the implementation of a child maltreatment preventive intervention delivered in primary care settings. The implementation process for each of these evidence-informed programs may present both similar and unique implementation challenges, and this is captured by the SIC.

The goal of the present study is to leverage the SIC database—the largest known repository of implementation process data from EIPs, including implementing sites and programs, their implementation process fidelity, and their achievement of program start-up and competency—to address the following questions:

1. Across evidence-informed programs, how much variability is there in achievement of (a) program start-up and (b) competency in program delivery?
2. To what extent is early implementation process fidelity associated with (a) program start-up and (b) competency in program delivery?

## Method

### Measures

#### Universal Stages of Implementation Completion (SIC)

The Universal SIC is a standardized measure of the implementation process spanning three implementation *phases*: Pre-Implementation, Implementation, and Sustainment [19]. Phases are divided into subsidiary SIC *stages*. The Pre-Implementation phase, which is of particular importance for the present study, is divided into three stages: Engagement (Stage 1), Consideration of Feasibility (Stage 2), and Readiness Planning (Stage 3). The Universal SIC stages are comprised of 46 implementation activities identified as being “common” across a range of EIPs [18]. Examples of activities (across all stages and phases) are presented in Table 1. Some programs choose to tailor their SIC by adding, modifying, or removing activities based on their individual implementation processes; however, the universal activities are largely represented across all SICs. With this pool of universal activities, SIC data can be combined across programs using the standard Universal SIC, providing an extensive data repository.

**Table 1:** SIC stages and phases with example activities

Phase	Stage		Example activities
1. Pre-Implementation	**1**	Engagement	Interest Indicated
			Initial Cost/Resource Info. Sent
	**2**	Consideration of Feasibility	Feasibility Questionnaire
			Program Champion Identified
	**3**	Readiness Planning	Community Partner Meeting
			Communication Plan
2. Implementation	**4**	Staff Hired and Trained	First Supervisor Selected
			Supervisor Training
	**5**	Fidelity Monitoring in Place	Fidelity Training Conducted
			Recording Equipment Tested
	**6**	Services and Consultation Begin	First Intake Assessment
			First Intervention Session
	**7**	Ongoing Program Delivery and Fidelity Monitoring	Supervisor Development Plan
			Achievement of Intervention Fidelity
3. Sustainment	**8**	Competency (certification)	Rated Competent for Sustainment

The program start-up implementation outcome is an activity in Phase 2, Stage 6. The competency implementation outcome is an activity in Phase 3, Stage 8. SIC activities in this table (e.g., Phase 2 Stage 5, Fidelity Monitoring in Place) refer to interven*tion fidelity*, as delivering the intervention as intended is an important component of *implementation process fidelity*

##### Data entry and validation

Programs partner with the SIC team to track new implementations. These programs agree for their deidentified data to be included in implementation research. Program purveyors leverage naturally occurring contacts to monitor an implementing sites’ progress. As each site advances through the implementation process, purveyors use the SIC website to report the date on which each activity was completed to a satisfactory standard. The SIC team works closely with purveyors to ensure data integrity. For example, possible data entry errors (e.g., program start-up precedes hiring of study staff) are identified by SIC staff during monthly validation checks and corrected if needed after discussion with purveyors.

##### Implementation process fidelity

The SIC yields two main scores: the *duration* of activity completion and the *proportion* of activities completed. The latter is the focus of the present investigation and reflects the proportion of activities completed in each phase (or stage). Activities are rated as “completed’ based on an operationalized definition to ensure that partially or inaccurately completed activities are not endorsed. The proportion score is computed by dividing the number of completed activities by the number of possible activities for the corresponding phase (or stage). These proportion scores reflect the level of implementation process fidelity for the respective implementation phase (or stage). Past research has indicated strong reliability for proportion scores of the Pre-Implementation phase (Phase 1; 15 activities; Rasch reliability = 0.81; Cronbach-equivalent test reliability = 0.89; site-level reliability = 0.72; [25]) and Implementation phase (Phase 2; 23 activities; Rasch reliability = 0.79; Cronbach-equivalent test reliability = 0.93; site-level reliability = 0.69). Phase 1 and Phase 2 proportion scores were available for all participating sites. The SIC most often is scored by phase; however, to address the present research questions, proportion scores also were calculated for the subsidiary Pre-Implementation stages: Stage 1 (Engagement, 2 Universal activities), Stage 2 (Feasibility, 4 Universal activities), and Stage 3 (Readiness Planning, 9 Universal activities).

##### Implementation outcomes

The SIC also captures two key implementation outcomes: achievement of program start-up and achievement of competency in program delivery. Each outcome is dichotomous (0 = *Not Achieved*, 1 = *Achieved*). Program start-up is achieved when a site first delivers services to clients/patients. Of sites that achieve program start-up, some will achieve competency in program delivery. Sites achieve competency when they can demonstrate that they deliver services from the EIP consistently and as intended. A site may have missing data for program start-up or both outcomes if its implementation is ongoing and the status of the outcome (not achieved or achieved) is not yet known.

### Sites and programs

The Universal SIC database included 1759 sites across 30 evidence-informed programs at the time of data retrieval (August 2020). Before analysis, some sites and EIPs were removed. One program was removed because its 207 sites completed all implementation activities in unison (i.e., there was no variability). Additionally, 92 sites were removed because they were “expansions” of established sites. Expansions reflect a need for increased service delivery capacity within an existing site, and as such, they do not reflect new implementation attempts. Finally, ongoing implementation attempts were eliminated (i.e., where start-up and competency status were unknown). The total sample of sites and programs varied across the two outcomes (i.e., achievement of program start-up and competency in program delivery) because for ongoing implementations, the start-up status is known prior to the competency status. The program start-up sample was 1287 sites across 27 evidence-informed programs, with the median program having 20 sites (*Mean* = 48, *Min*. = 1, *Max*. = 364). From this, *n* = 182 sites without a known status for achieving competency were removed. This resulted in *n* = 1105 sites across 19 evidence-informed programs, with the median program having 25 sites (*Mean* = 58, *Min*. = 2, *Max*. = 359). Table 2 provides a summary of the complete sample of 27 EBIs including program focus, population, and setting.

**Table 2:** Program and site sample sizes and start-up rates summarized by key program characteristics across 27 different evidence-informed programs

Program		Sample size		Start-up rate^a^		
Characteristic	Type	Programs	Sites	Mean	Min	Max
Focus	Behavioral Health	15	695	0.61	0.13	1.00
	Child Welfare	5	476	0.63	0.07	1.00
	Other^*b*^	3	99	0.38	0.00	1.00
	Criminal Justice	2	7	1.00	1.00	1.00
	Substance Use	2	10	0.61	0.22	1.00
Population	Adult	12	310	0.58	0.00	1.00
	Child	9	830	0.51	0.13	1.00
	Organizational	4	124	0.89	0.77	1.00
	Family	2	23	0.80	0.60	1.00
Setting	Child Welfare	6	203	0.67	0.07	1.00
	Education	5	214	0.50	0.13	0.79
	Justice	5	162	0.75	0.16	1.00
	Healthcare	4	105	0.37	0.00	1.00
	Community	3	14	0.74	0.22	1.00
	Multiple^*c*^	2	480	0.45	0.26	0.65
	Substance Use	2	109	0.88	0.77	0.98

To ensure that individual programs are not identifiable, program characteristics are not reported on a program-by-program basis. Instead, for each program characteristic (i.e. focus, population, and setting), the number of programs and sites is summarized, along with the start-up rate for those programs

^a^Descriptive start-up rate at program level (i.e., computed as the average of each program’s average start-up rate across its sites)

^b^Includes programs focused on physical health and child welfare prevention, collapsed to prevent identification

^c^Programs that are implemented in more than one setting

### Data analysis strategy

The two outcomes—achievement of program start-up and competency in program delivery—were dichotomous and modeled according to a Bernoulli distribution (logit link) with adaptive quadrature estimation. The data were structured with sites (level-1) nested within evidence-informed programs (level-2). Specifically, each site attempted implementation of only one EIP. Nesting was addressed using mixed-effects models [26] implemented in SuperMix Version 2.1 [27]. The competency outcome was available for only a modest number of EIPs (*n* = 19). However, simulation studies with as few as 10 upper-level units have indicated that level-1 regression coefficients (and associated SEs) were estimated accurately, though there was notable bias for upper-level variance components [28, 29].

For the program start-up outcome, two groups of models were performed. The first evaluated the proportion score for the overall Pre-Implementation *phase*, and the second evaluated the proportion score for each individual Pre-Implementation *stage*. The latter included separate models for activities completed in Engagement (Stage 1), Consideration of Feasibility (Stage 2), and Readiness Planning (Stage 3). For each model, the focal proportion score was centered around its grand mean prior to entry, and the corresponding random effect specification was based on the likelihood ratio test. For the achievement of competency in program delivery outcome, which is later in the implementation process, the model was extended to include implementation process fidelity in the Implementation phase. Of note, completing any activity in the Implementation phase meant—by definition—having completed at least one activity in the prior Pre-Implementation phase. Because of this, it was not viable to enter a main effect for Implementation proportion. Instead, the model only included a main effect for Pre-Implementation proportion (as described for the program start-up outcome) and an interaction between Pre-Implementation and Implementation proportions. As above, each term was centered prior to entry, but because of the reduced number of EIPs with sites achieving the competency outcome, random effects were not evaluated for the predictors.

## Results

### Descriptive statistics

Table 3 presents descriptive statistics for the proportion of activities completed in Pre-Implementation—including the subsidiary Stages 1, 2, and 3—as well as the Implementation phase, organized by implementation outcome status (i.e., discontinued prior to start-up, achieved start-up, discontinued prior to competency, or achieved competency). Across the N = 1,287 sites with a known status for program start-up, the rate of start-up was 35% (*N* = 455). The average across evidence-informed *programs* was nearly twice as high at 62% (*Median* = 68%, *Min.* = 0%, *Max.* = 100%). The discrepancy between sites and programs reflects considerable variability across programs, both in the number of sites and the rates of achieving start-up. Table 2 provides the average start-up rates by EIP focus, population, and setting.

**Table 3:** Descriptive statistics for implementation process fidelity by implementation outcome status

		Stage 1	Stage 2	Stage 3	Phase 1	Phase 2^a^
Implementation Status	*N*	Mean (*SD*)	Mean (*SD*)	Mean (*SD*)	Mean (*SD*)	Mean (*SD*)
Start-Up Status	1287	0.72 (0.37)	0.38 (0.45)	0.34 (0.42)	0.41 (0.39)	
Discontinued	832	0.59 (0.39)	0.11 (0.26)	0.06 (0.16)	0.15 (0.18)	
Achieved	455	0.97 (0.13)	0.89 (0.21)	0.85 (0.23)	0.88 (0.19)	
Competence Status	1105	0.69 (0.38)	0.30 (0.41)	0.24 (0.38)	0.33 (0.36)	0.22 (0.38)
Discontinued	943	0.63 (0.39)	0.19 (0.35)	0.14 (0.28)	0.23 (0.28)	0.10 (0.26)
Achieved	162	0.99 (0.06)	0.89 (0.22)	0.87 (0.22)	0.89 (0.17)	0.91 (0.12)

Stage 1 = Engagement. Stage 2 = Consideration of Feasibility. Stage 3 = Readiness Planning. Phase 1 = Pre-Implementation. Phase 2 = Implementation

^a^Sites with a known end-status on the start-up outcome (i.e., they have either achieved start-up or discontinued) may still be ongoing during Phase 2. Since ongoing sites may go on to complete more activities during Phase 2 before achieving competence or discontinuing, the proportion scores for ongoing sites are skewed negative in a manner that misrepresents those sites’ ultimate Phase 2 implementation process fidelity. Therefore, Phase 2 proportion scores are only reported for the subset with a known end-status for achieving competency

Across the subset of *N* = 1,105 sites with a known status for competency, only 15% achieved competency (*N* = 162). When considering the average across 19 evidence-informed programs, the rate was comparable at 17% (*Median* = 5%, *Min.* = 0%, *Max* = 67%). Of the sites that did not achieve program start-up (*n* = 832 of 1287, 65%), 96% (*n* = 798) discontinued in the Pre-Implementation phase (i.e., Stages 1, 2 or 3), with 62% (*n* = 518) discontinuing in Stage 1, 18% (*n* = 149) in Stage 2, and 16% (*n* = 131) in Stage 3. Of the sites that did not achieve competency (*n* = 943 of 1105, 85%), 85% (*n* = 798) discontinued during Pre-Implementation. In contrast, of sites that completed the Pre-Implementation phase, 93% achieved program start-up and 53% achieved competency.

### Prediction models

#### Program start-up and competency by program

To estimate the overall rate of program start-up and competency in program delivery across *sites*, an initial unconditional model was performed that did not include programs. This was followed by an unconditional two-level mixed-effects regression model that nested sites (level-1) within programs (level-2). Across all *sites*, the overall predicted probability of achieving start-up was 35% (95% CI [33%, 38%]; β = -0.60, *SE* = 0.058, *z* = 10.41, *p* < 0.001). When considering the program being implemented, the average predicted probability of start-up was nearly twice as high at 64% (95% CI [42%, 82%], β = 0.59, *SE* = 0.47, *z* = 1.24, *p* = 0.215), and 57% of the total variance in start-up was attributable to the program (τ_π_ = 4.33; with the remaining 43% attributable to sites). For achievement of competency, across all sites, the overall predicted probability was 15% (95% CI [13%, 17%]; β = -1.76, *SE* = 0.085, *z* = -20.71, *p* < 0.001). When considering the program being implemented, the average predicted probability of competency was lower at 8% (95% CI [3%, 20%], β = -2.41, *SE* = 0.09, *z* = -4.66, *p* < 0.001), and 51% of the total variance in competency was attributable to the program (τ_π_ = 3.44; with the remaining 49% attributable to sites). The differing estimates—when only considering sites versus when also considering programs—are attributable to variability in the number of sites per program and the program-specific rates of start-up and competency.

#### Implementation process fidelity and program start-up

##### Phase proportion

The two-level unconditional model for program start-up was extended to include the proportion of activities completed in the Pre-Implementation phase (i.e., across Stages 1–3). Completion of Pre-Implementation activities was significantly and positively associated with achieving program start-up (*p* < 0.001; Table 4), and the effect of Pre-Implementation proportion did not vary significantly by program. As an example, for sites completing 75% versus 25% of Pre-Implementation activities, the predicted probability of program start-up would be 95% versus 2%.

**Table 4:** Results of mixed-effects logistic regression models with SIC proportion scores by phase and stage predicting program start-up status

Outcome	Model	Predictor		Fixed effects					Variance components^b,c^	
				β	*SE*	*p*	95% CI		*Var*	*SE*
Start-Up^d^										
	*Phase 1*									
		Intercept		-1.73	0.74	.019	[-3.17, -0.29]		8.86	3.83
		Proportion^a^		13.78	1.10	< .001	[11.63, 15.94]			
	*Stage 1*									
		Intercept		-0.97	1.09	.373	[-3.10, 1.16]		8.70	6.08
		Proportion^a^		8.33	3.39	.014	[1.68, 14.98]		63.19	59.55
	*Stage 2*									
		Intercept		-1.51	0.38	< .001	[-2.25, -0.77]		1.92	0.87
		Proportion^a^		7.87	0.47	< .001	[6.94, 8.79]			
	*Stage 3*									
		Intercept		-1.23	0.83	.139	[-2.87, 0.40]		11.98	5.23
		Proportion^a^		13.33	1.11	< .001	[11.16, 15.50]			
Competence^e^										
	*Phases 1 & 2*									
		Intercept		-4.53	0.45	< .001	[-5.41, -3.65]		1.1138	0.7959
		Phase 1^a^		3.07	0.83	< .001	[1.46, 4.69]			
		Phases 1 × 2^a^		8.22	1.42	< .001	[5.45, 11.00]			

^a^Proportion scores range from 0.00 to 1.00 and were grand mean centered prior to entry, thus the intercept reflects the log-odds of program start-up for an average proportion in the respective phase or stage

^b^Site-level variance component estimates are not available for the Bernoulli outcome distribution and, as such, the reported estimates are limited to program-level variance

^c^Variance components for the proportion predictor specified based on the likelihood ratio test

^d^The sample for this regression include *N* = 1287 sites with a known end-status (discontinued or achieved start-up) for program start-up

^e^The sample for this regression include *N* = 1105 sites with a known end-status (discontinued or achieved competence) for competency

##### Stage proportion

Pre-Implementation proportion (i.e., phase proportion) was removed, and separate models were performed to evaluate each *stage-specific* proportion score. In each case, stage proportion was significantly associated with achieving program start-up, with a higher proportion of completed activities associated with a higher log-odds of program start-up (*p*s < 0.015; Table 4).

**Fig. 1:**
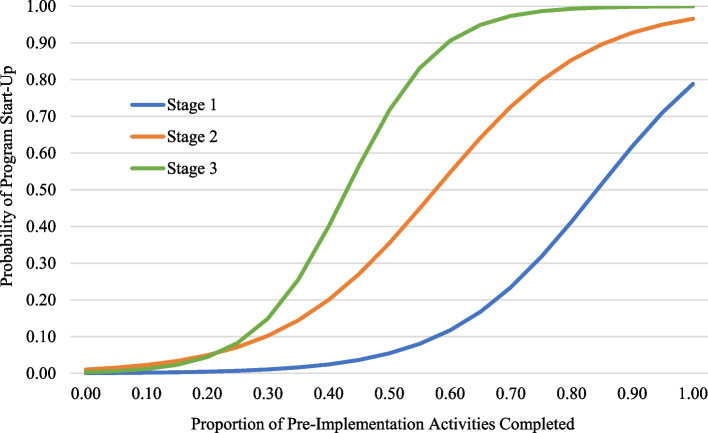
The predicted probability of achieving program start-up based on the proportion of Universal SIC activities completed in a given stage of the Pre-Implementation phase. *Note*. For each of the three stage proportion scores, the predicted probabilities are based on a separate mixed-effects logistic regression model

Although the direction of the association was the same across Stage 1, Stage 2, and Stage 3, as illustrated in Fig. 1, the shape of that association varied by stage. With a low level of activity completion in a given stage, such as 10%, the predicted probability of achieving program start-up was low regardless of the stage: < 1% for activities completed in Stage 1, 2% for Stage 2, and 1% for Stage 3. In contrast, for moderate activity completion, such as 50%, the predicted probability of start-up varied by stage: 5% for activities completed in Stage 1, 35% for Stage 2, and 72% for Stage 3. For 100% activity completion, the probability of program start-up was high for activities completed in Stages 2 and 3, at 97% and > 99%, but somewhat lower for activities completed in Stage 1, at 79%.

It is important to note that these predicted probabilities reflect averages across all EIPs. However, with 57% of the outcome variance attributable to program, there was strong evidence of variability from program to program. Furthermore, for Stage 1 only (i.e., not Stages 2 or 3), the association between proportion and start-up varied significantly across programs. To illustrate this, Empirical Bayes residuals were used to calculate the specific probability of start-up for each program (the complete table of which is available upon request). In Stage 3, if a site completed all activities, the probability of start-up was ≥ 95% for all programs. Stage 2 was similar, with completion of all activities leading to a ≥ 84% probability of start-up for all but one program. For Stage 1, that probability decreased to 79%; however, for over one-fifth of programs—which represented nearly *half of all sites*—the probability of achieving program start-up was less than 40%. Stated differently, for a large number of sites, completing all activities in Stage 1 was not sufficient for a high probability of program start-up. Thus, across stages, the likelihood of program start-up increased as sites completed more implementation activities. However, for Stage 1, completion of all activities was associated with a somewhat lower likelihood of start-up compared to completion of all activities in Stages 2 or 3. Furthermore, only Stage 1 showed evidence of variability across programs in this association, with some programs being more likely to achieve program start-up at high Engagement activity completion than others.

#### Implementation process fidelity and competency in program delivery

**Fig. 2:**
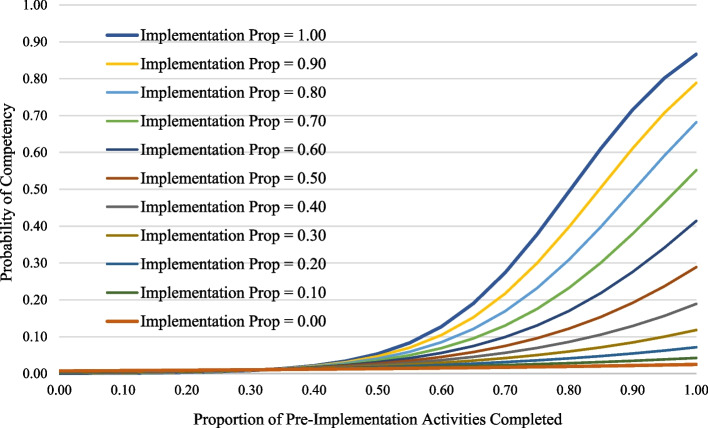
The predicted probability of achieving competency based on the proportion of Universal SIC activities completed in Phase 1: Pre-Implementation by Phase 2: Implementation Proportion. *Note*. This figure graphically represents Phase 1 and Phase 1 × 2 proportion scores predicting site competency status

The two-level unconditional model for competency in program delivery was extended to include the proportion of activities completed in the Pre-Implementation phase and the interaction between Pre-Implementation and Implementation proportion. Pre-Implementation proportion was significantly and positively associated with achieving competency (*p* < 0.001, see Table 4). The interaction between Pre-Implementation and Implementation proportion scores was also positive and significant (*p* < 0.001; see Table 4). This reflects a differential effect of Implementation proportion depending on the level of Pre-Implementation proportion. As illustrated in Fig. 2, when not considering the influence of Implementation fidelity (i.e., Implementation proportion = 0.00), the full range of the Pre-Implementation fidelity (i.e., 0.00 versus 1.00) increased the probability of achieving competency from < 1% to 7%. Generally, as implementation process fidelity increased during Implementation, the probability of achieving competence increased above the level expected from Pre-Implementation alone. However, Pre-Implementation fidelity played an influential role. If it was low (e.g., Pre-Implementation = 0.10), the probability of achieving competency was low regardless of the level of Implementation fidelity—even if it was 1.00. In contrast, if Pre-Implementation fidelity was high (e.g., Pre-Implementation = 0.90), the probability of achieving competency depended on the level of Implementation fidelity. This ranged from 7%, if Implementation fidelity was low (i.e., 0.00), to 87%, if Implementation fidelity was high (i.e., 1.00). Thus, Implementation fidelity had a significant differential effect depending on the level of Pre-Implementation process fidelity.

## Discussion

This study provides empirical evidence supporting the critical value of Pre-Implementation for the successful implementation of EIPs. Not only does Pre-Implementation support the start-up of a new program, but programs that start-up after a poor Pre-Implementation process are unlikely to achieve competency in program delivery. Just as the measurement of *intervention* fidelity (the extent to which the intervention is delivered as intended) can promote positive treatment outcomes, positive implementation outcomes may be supported by measuring sites’ *implementation process* fidelity. A repository of data collected using the Universal SIC provided an unprecedented opportunity to examine the relationship between implementation process and outcomes across a range of programs, using a shared measure of implementation process fidelity.

Across over a thousand implementation attempts, most implementing sites—upwards of 85%—discontinued before achieving program start-up or competency in program delivery. Notably, different EIPs achieved those implementation milestones at different rates. Importantly, when considering the nesting of sites within evidence-informed programs—rather than sites alone—the average predicted probability of achieving start-up nearly doubled. This reflects the presence of programs with many implementing sites that have overall lower rates of achieving start-up. Despite considerable variation across programs, the completion of SIC-defined activities—that is, high implementation process fidelity—reliably predicted positive implementation outcomes for a range of EIPs. Specifically, across 27 programs and 1287 sites, Pre-Implementation fidelity was positively and significantly associated with achieving program start-up for newly adopting sites. In addition, across 1105 individual implementation attempts for 19 evidence-informed programs, SIC-derived Pre-Implementation and Implementation fidelity scores were positive and significant predictors of sites achieving competency in program delivery. Critically, engagement with the implementation process was only a potent predictor of positive implementation outcomes when sites reported high implementation process fidelity during the early Pre-Implementation phase.

### Pre-Implementation: a critical period in the implementation process

When a site engages with the implementation process, the ultimate goal is for the desired services to reach the target population. Traditionally, implementers and those who fund them might prioritize the activities in the SIC-defined Implementation phase, jumping ahead to serving clients, and this might vary by setting. For instance, although not the focus of analyses, in the current sample EIPs implemented in Education or Healthcare settings averaged less time spent in Pre-Implementation (mean 5.9 and 4.2 months respectively) than those is Justice or Child Welfare settings (mean = 13.4 and 9.0 months respectively). However, the present study demonstrates that sites with strong implementation process fidelity in the Implementation phase still have low probabilities of achieving competency in program delivery, which is necessary to sustain the program, if they had poor Pre-Implementation process fidelity (Fig. 2). Given that 85% of implementing sites discontinue before achieving competency, activities early in the implementation process appear to be critical for successful implementation. This might be particularly true for complex interventions; of the EIPs included in the current analyses, most represent programs implemented in system settings such as child welfare, education, criminal justice, healthcare, or combined settings (Table 2), where implementation activities necessitate involvement of multiple levels of leadership, providers, and community partners. To support positive implementation outcomes, it is necessary to: 1) to support sites’ engagement in early implementation activities and 2) understand the relationship between those outcomes and early implementation process. The SIC provides us with the tools to measure and evaluate such activity: proportion scores from Stages 1, 2, and 3 of the Pre-Implementation phase.

### The importance of Pre-Implementation stages

Each of the three Pre-Implementation stages are positively, significantly, and independently associated with program start-up. Although the Pre-Implementation phase (across all of the first three stages) typically stands alone (e.g., [16, 21]) and was shown in the current study to be predictive of program start-up, the relative importance of each stage was remarkable. This provides further evidence that each of the three stages measures different aspects of Pre-Implementation fidelity [25]. Additionally, and more importantly, by understanding the unique and important role of Engagement, Consideration of Feasibility, and Readiness Planning activities during Pre-Implementation, adopters can better understand both how to focus their implementation efforts and the importance of maintaining a thorough implementation process. Thus, not unlike measures of *intervention* fidelity that assess the delivery of the intervention in order to achieve positive clinical outcomes, the SIC as a measure of *implementation process* fidelity assesses the completion of activities that contribute to positive implementation outcomes.

The relative value of each Pre-Implementation stage is not surprising. Results showed that activities completed during the Readiness Planning stage were most impactful in supporting program start-up. The importance of completing Readiness activities is well-cited in the implementation science literature [30–33]. In fact, a number of standardized instruments exist for measuring organizational readiness to implement new programs (e.g., ORIC, ORC, ORCA; [33–35]), and many evidence-informed programs come with guidance or technical assistance for sites in how to conduct Readiness activities. On the other hand, even high levels of activity completion for the Engagement stage were associated with comparatively meager chances of achieving program start-up. Thus, supporting the field’s plea for implementation strategies that facilitate successful program start-up [36, 37], the present results illustrate that Engagement—informing a site about a program, engaging them in discussions about program fit, and providing them with program materials—is insufficient for successful adoption.

Of note, for sites that completed a large proportion of Engagement activities, the probability of achieving program start-up was surprisingly high (79%) compared to prior research, where this probability was estimated at 50% or less [15]. This discrepancy is largely due to sample size considerations. Prior work included fewer evidence-informed programs and, as such, each site was considered to be independent, making large programs disproportionately influential. In contrast, the present study was able to nest sites within programs, which meant that each evidence-informed program contributed an equal amount of information. Indeed, in the current analyses, program specific estimates were consistent with prior research: specifically, for half of all sites, the predicted probability of achieving program start-up at 100% activity completion for the Engagement stage was less than 40%. Regardless, the current outcome suggests the opportunity for sharing of knowledge across programs to increase successful rates of engagement.

### Discontinuing implementation

Importantly, analyses from this study highlight the high degree of discontinuation (62%) among sites attempting to implement a new EIP—the large majority discontinuing in Pre-Implementation (96%). Yet, of those that do complete Pre-Implementation, nearly all (93%) achieve program start-up, providing further support for the value of completing Engagement, Consideration of Feasibility, and Readiness activities with quality when preparing sites for successful program start-up. This suggests that implementation interventions should be developed to support the completion of Pre-Implementation activities. To develop such interventions, there are two important considerations from the present findings. One is that Readiness activities offered the most robust contribution to program start-up (followed by Consideration of Feasibility, then Engagement). However, the other is that the majority of sites discontinued in the Engagement stage. Because of this, interventions targeting the Engagement stage—such as decision-making to adopt evidence-informed programs—although as noted above are insufficient alone, could be particularly useful. There are a range of web-based tools that provide sites with menus of evidence-informed programs for implementation (e.g., Administration for Community Living [38]; California Evidence-Based Clearinghouse for Child Welfare [39]; Evidence-Based Cancer Control Programs [40]; youth.gov [41]). However, low-burden engagement interventions are less readily available. Such interventions could aid sites during early implementation by providing context-specific guidance. Methods such as multi-criteria decision analysis tools provide a promising avenue for assisting stakeholders in identifying the evidence-informed programs that are most likely to yield successful engagement [42]. Recent analyses suggest that when developing such tools, decision-makers report wanting to know whether or not the program provides support with the implementation process as an essential piece of information for their consideration before deciding whether or not to complete engagement [43]. An operationalized implementation process, such as that provided by the SIC, could aid decision-makers in understanding what activities are necessary to implement the program successfully and to identify what implementation supports are necessary for the site to complete each activity.

### Limitations

The SIC was designed to measure the implementation process for behavioral (e.g., child externalizing problems) and physical healthcare (e.g., hypertension) programs; therefore, conclusions from the present study are constrained to these types of programs. An additional limitation is that the outcomes of interest, program start-up and competency, are only two of several possible outcomes for the implementation process. For instance, these analyses did not explore the influence of Pre-Implementation on long-term sustainment of the intervention. It is possible that early features of the implementation process may influence distal implementation outcomes, and these are ripe avenues for future research (see [16]). Next, the present analyses evaluated each Pre-Implementation stage as a stand-alone predictor of program start-up. This was important for addressing the primary study aims but did not extend to inter-relationships in Pre-Implementation fidelity across Stages 1, 2, and 3. Additionally, proportion scores for the three Pre-Implementation stages did not have accompanying psychometric data because they were based on a modest number of activities. However, there is evidence that the Pre-Implementation phase—rather than individual stages—forms a distinct dimension, with activities sufficient for reliable measurement. The present focus on stage scores was for the purpose of addressing targeted research questions for this study. The results should be interpreted in the context of this limitation; however, it is important to note that the stage-specific SIC activities reflect the population—rather than a small sub-sample—of universal implementation activities. Finally, the number of EIPs was modest for mixed-effects regression models: 27 for program start-up and 19 for competency in program delivery. Additionally, some programs had a small number of sites—for example, two programs had only one implementing site. However, prior simulation studies [28, 29] have supported the accuracy of the model parameters for the primary research questions, though the program-level variance components should be interpreted with caution.

## Conclusions

The present study leveraged the largest known repository of independent implementation attempts to investigate the association between implementation process fidelity and achievement of two implementation milestones: program start-up and competency in program delivery. There were three main conclusions: First, although rates of achieving implementation milestones varied significantly across EIPs, they generally were quite low. Second, the probability of achieving those milestones was significantly higher when a site maintained high implementation process fidelity during the Pre-Implementation stages. Finally, high implementation process fidelity during the Implementation phase was not sufficient to produce positive implementation outcomes—that is, a newly adopting site was more likely to achieve competent delivery of the desired program if they had high Pre-Implementation *and* Implementation fidelity. This indicates that Pre-Implementation is a critical period in the implementation process. Further research is underway by the investigative team and others in the field (e.g., [30–33]) to develop implementation interventions and support tools to improve Pre-Implementation fidelity. Furthermore, funders and allies of evidence-informed program adoption should accommodate the timelines and resources necessary for sites to conduct strong Pre-Implementation. In so doing, sites will be well-positioned to achieve program start-up and competency in program delivery, increasing the number of evidence-informed programs provided to patients and communities in need.

## Data Availability

The datasets used and/or analyzed during the current study are available from the corresponding author on reasonable request.

## Abbreviations

EIP: Evidence informed program
SIC: Stages of implementation completion
EPIS: Exploration, preparation, implementation, and sustainment
